# Supplementary material for: Discovery and application of insertion-deletion (INDEL) polymorphisms for QTL mapping of early life-history traits in Atlantic salmon
Source: BMC Genomics. 2010 Mar 8;11:156. doi: 10.1186/1471-2164-11-156 (PMC2838853; doi:10.1186/1471-2164-11-156)
Supplement: Additional file 2 — Information on developed 76 locus single-run INDEL panel in Atlantic salmon. Information on fluorescence labeling, primer concentrations, PCR pooling and links to alignments, INDEL motifs and GENESCAN (Burge and Karlin 1997) predictions of genes/exons are available in html format. [file 1471-2164-11-156-S2.ZIP › Additionalfile2/table8921.html]

Contig Alignment

```
Base	A B C D E F   
1	. C . . . .    
2	. T . . . .    
3	. C . . . .    
4	. A . . . .    
5	. A . . . .    
6	. T . . . .    
7	. C . . . .    
8	. T . . . .    
9	. C . . . .    
10	. A . . . .    
11	. G G . . .    
12	. G G . . .    
13	. A A . . .    
14	. A A . . .    
15	. G G . . .    
16	. C C . . .    
17	. G G . . .    
18	. T T . . .    
19	. T T . . .    
20	. G G . . .    
21	. A A . . .    
22	. T T . . .    
23	. G G . . .    
24	. A A . . .    
25	. T T . . .    
26	. G G . . .    
27	. T T . . .    
28	. T T . . .    
29	. C C . . .    
30	. T T . . .    
31	. A A . . .    
32	. G G . . .    
33	. A A . . .    
34	. G G . . .    
35	. G G . . .    
36	. A A . . .    
37	. C C . . .    
38	. G G . . .    
39	. A A . . .    
40	. G G . . .    
41	. T T . . .    
42	. T T . . .    
43	. T T . . .    
44	. G G . . .    
45	. T T . . .    
46	. C C . . .    
47	. T T . . .    
48	. G G . . .    
49	. A A . . .    
50	. G G . . .    
51	. A A . . .    
52	. C C . . .    
53	. C C . . .    
54	. A A . . .    
55	. A A . . .    
56	. A A . . .    
57	. G G . . .    
58	. A A . . .    
59	. C C . . .    
60	. A A . . .    
61	. G G . . .    
62	. A A . . .    
63	. C C . . .    
64	. A A . . .    
65	. T T . . .    
66	. G G . . .    
67	. T T . . .    
68	. C C . . .    
69	. A A . . A    
70	. A A . . A    
71	. A A . . A    
72	. T T . . T    
73	. T T . . T    
74	. T T . . T    
75	. A A . . A    
76	. T T . . T    
77	. T T . . T    
78	. T T . . T    
79	. C C . . C    
80	. A A . . A    
81	. G G . . G    
82	. C C . . C    
83	. A A . . A    
84	. C C . . C    
85	. T T . . T    
86	. G G . . G    
87	. A A . . A    
88	. C C . . C    
89	. C C . . C    
90	. A A . . A    
91	. T T . . T    
92	. C C . . C    
93	. C C . . C    
94	. A A . . A    
95	. C C . . C    
96	. T T . . T    
97	. G C . . C    
98	. T T . . T    
99	. G G . . G    
100	. C C . . C    
101	. A A . . A    
102	. T T . . T    
103	. C C . . C    
104	. A A . . A    
105	. A A . . A    
106	. C C . . C    
107	. T T . . T    
108	. C C . . C    
109	. C C . . C    
110	. A A . . A    
111	. T T . . T    
112	. G G . . G    
113	. T T . . T    
114	. T T . . T    
115	. A A . . A    
116	. T T . . T    
117	. G G . . G    
118	. A A . . A    
119	. A A . . A    
120	. G G . . G    
121	. T T . . T    
122	. G G . . G    
123	. G G . . G    
124	. A A . . A    
125	. C C . . C    
126	. A A A . A    
127	. C C C . C    
128	. C C C . C    
129	. A A A . A    
130	. A A A . A    
131	. C C C . C    
132	. C C C . C    
133	. C C C . C    
134	. C C C . C    
135	. C C C . C    
136	. A A A . A    
137	. C C C . C    
138	. G G G . G    
139	. T T T . T    
140	. C C C . C    
141	. A A A . A    
142	. C C C . C    
143	. C C C . C    
144	. A A A . A    
145	. A A A . A    
146	. C C C . C    
147	. C C C . C    
148	. C C C . C    
149	. C C C . C    
150	. C C C . C    
151	. T T T . T    
152	. C C C . C    
153	. A A A . A    
154	C C C C C C    
155	C C C C C C    
156	C C C C C C    
157	T T T T T T    
158	T C C C T C  2	 transition  
159	T T T T T T    
160	T T T T T T    
161	C C C C C C    
162	A A A A A A    
163	A A A A A A    
164	G G G G G G    
165	A A A A A A    
166	A A A A A A    
167	G G G G G G    
168	C C C C C C    
169	C C C C C C    
170	T T T T T T    
171	T T T T T T    
172	A A A A A A    
173	C C C C C C    
174	T T T T T T    
175	T T T T T T    
176	G G G G G G    
177	A A A A A A    
178	C C C C C C    
179	T T T T T T    
180	A A A A A A    
181	A A A A A A    
182	T T T T T T    
183	G G G G G G    
184	C C C C C C    
185	T T T T T T    
186	G G G G G G    
187	A A A A A A    
188	A A A A A A    
189	T T T T T T    
190	C C C C C C    
191	C C C C C C    
192	C C C C C C    
193	A A A A A A    
194	A A A A A A    
195	A A A A A A    
196	T T T T T T    
197	G G G G G G    
198	G G G G G G    
199	A A A A A A    
200	C C C C C C    
201	C C C C C C    
202	A A A A A A    
203	T T T T T T    
204	T T T T T T    
205	A A A A A A    
206	G G G G G G    
207	C C C C C C    
208	C C C C C C    
209	C C C C C C    
210	C C C C C C    
211	A A A A A A    
212	T T T T T T    
213	A A A A A A    
214	C C C C C C    
215	T T T T T T    
216	C C C C C C    
217	C C C C C C    
218	T T T T T T    
219	T T T T T T    
220	G G G G G G    
221	G G G G G G    
222	C C C C C C    
223	A A A A A A    
224	T T T T T T    
225	T T T T T T    
226	T T T T T T    
227	T T T T T T    
228	A A A A A A    
229	T T T T T T    
230	G G G G G G    
231	T T T T T T    
232	T T T T T T    
233	T T T T T T    
234	A A A A A A    
235	T T T T T T    
236	T T T T T T    
237	T T T T T T    
238	G G G G G G    
239	A A A A A A    
240	A A A A A A    
241	A A A A A A    
242	C C C C C C    
243	A A A A A A    
244	A A A A A A    
245	T T T T T T    
246	T T T T T T    
247	G G G G G G    
248	G G G G G G    
249	A A A A A A    
250	T T T T T T    
251	G G G G G G    
252	A A A A A A    
253	T T T T T T    
254	A A A A A A    
255	A A A A A A    
256	C C C C C C    
257	A A A A A A    
258	A A A A A A    
259	T T T T T T    
260	T T T T T T    
261	T T T T T T    
262	T T T T T T    
263	T T T T T T    
264	G G G G G G    
265	C C C C C C    
266	A A A A A A    
267	T T T T T T    
268	A A A A A A    
269	A A A A A A    
270	C C C C C C    
271	C C C C C C    
272	T T T T T T    
273	C C C C C C    
274	A A A A A A    
275	G G G G G G    
276	T T T T T T    
277	G G G G G G    
278	G G G G G G    
279	G G G G G G    
280	C C C C C C    
281	A A A A A A    
282	A A A A A A    
283	G G G G G G    
284	G G G G G G    
285	T T T T T T    
286	G G G G G G    
287	A A A A A A    
288	A A A A A A    
289	C C C C C C    
290	C C C C C C    
291	A A A A A A    
292	C C C C C C    
293	T T T T T T    
294	T T T T T T    
295	C A A A A A    
296	C C C C C C    
297	C C C C C C    
298	A A A A A A    
299	T T T T T T    
300	G G G G G G    
301	T T T T T T    
302	G G G G G G    
303	G G G G G G    
304	T T T T T T    
305	C C C C T C    
306	T T T T T T    
307	G G G G G G    
308	A A A A A A    
309	A A A A A A    
310	C C C C C C    
311	C C C C C C    
312	T T T T T T    
313	A A A A A A    
314	T T T T T T    
315	C C C C C C    
316	C C C C C C    
317	G G G G G G    
318	A A A A A A    
319	T T T T T T    
320	C C C C C C    
321	A A A A A A    
322	C C C C C C    
323	T T T T T T    
324	A A A A A A    
325	C C C C C C    
326	T T T T T T    
327	G G G G G G    
328	A A A A A A    
329	T T T T T T    
330	T C C C C C    
331	T T T T T T    
332	A A A A A A    
333	C C C C C C    
334	T T T T T T    
335	G G G G G G    
336	G G G G G G    
337	A A A A A A    
338	A A A A A A    
339	T T T T T T    
340	G G G G G G    
341	G G G G G G    
342	T T T T T T    
343	T T T T T T    
344	A A A A A A    
345	G G G G G G    
346	A A A A A A    
347	G G G G G G    
348	G G G G G G    
349	T T T T T T    
350	C C C C C C    
351	G G G G G G    
352	A A A A A A    
353	T T T T T T    
354	T T T T T T    
355	T T T T T T    
356	G G G G G G    
357	G G G G G G    
358	A A A A A A    
359	A A A A A A    
360	T T T T T T    
361	G G G G G G    
362	C C C C C C    
363	A A A A A A    
364	G G G G G G    
365	A A A A A A    
366	C C C C C C    
367	T T T T T T    
368	G G G G G G    
369	T T T - T -  2	 indel  
370	G G G - G -  2	 indel  
371	C C C C C C    
372	A A A A A A    
373	G G G G G G    
374	C C C C C C    
375	A A A A A A    
376	G G G G G G    
377	T T T T T T    
378	T T T T T T    
379	C C C C C C    
380	T T T T T T    
381	C C C C C C    
382	C C C C C C    
383	C C C C C C    
384	A A A A A A    
385	G G G G G G    
386	C C C C C C    
387	C C C C C C    
388	A A A A A A    
389	A A A A A A    
390	T T T T T T    
391	T T T T T T    
392	T T T T T T    
393	C C C C C C    
394	A A A A A A    
395	T T T T T T    
396	T T T T T T    
397	G G G G G G    
398	A A A A A A    
399	A A A A A A    
400	C C C C C C    
401	T T T T T T    
402	G G G G G G    
403	T T T T T T    
404	T T T T T T    
405	G G G G G G    
406	T T T T T T    
407	C C C C C C    
408	T T T T T T    
409	T T T T T T    
410	T T T T T T    
411	C C C C C C    
412	C C C C C C    
413	C C C C C C    
414	C C C C C C    
415	A A A A A A    
416	A A A A A A    
417	T T T T T T    
418	A A A A A A    
419	C C C C C C    
420	G G G G G G    
421	G G G G G G    
422	G G G G G G    
423	T T T T T T    
424	T T T T T T    
425	G G G G G G    
426	A A A A A A    
427	T T T T T T    
428	T T T T T T    
429	T T T T T T    
430	T T T T T T    
431	T T T T T T    
432	G G G G G G    
433	G G G G G G    
434	A T T T T T    
435	. A A A A A    
436	. A A A A A    
437	. T T T T T    
438	. T T T T T    
439	. C C C C C    
440	. C C C C C    
441	. C C C C C    
442	. T T T T T    
443	. T T T T T    
444	. A A A A A    
445	. T T T T T    
446	. G G G C G    
447	. C C C C C    
448	. C C C C C    
449	. T T T T T    
450	. T T T T T    
451	. T T T T T    
452	. C C C C C    
453	. A A A A A    
454	. T T T G T    
455	. G G G G G    
456	. C C C C C    
457	. A A A A A    
458	. G G G G G    
459	. T T T T T    
460	. T T T T T    
461	. A A A A A    
462	. T T T T T    
463	. G G G C G    
464	. C C C C C    
465	. C C C C C    
466	. T T T T T    
467	. T T T T T    
468	. G G G G G    
469	. T T T G T    
470	. G G G G G    
471	. C C C C C    
472	. T T T T T    
473	. G G G G G    
474	. C C C C C    
475	. T T T T T    
476	. A A A A A    
477	. T T T T T    
478	. G G G G G    
479	. T T T T T    
480	. G G G G G    
481	. T T T T T    
482	. T T T T T    
483	. T T T T T    
484	. A A A A A    
485	. T T T T T    
486	. T T T T T    
487	. G G G G G    
488	. T T T T T    
489	. T T T T T    
490	. T T T T T    
491	. A A A A A    
492	. A A A A A    
493	. G G G G G    
494	. T T T T T    
495	. T T T T T    
496	. G G G G G    
497	. C C C C C    
498	. T T T T T    
499	. G G G G G    
500	. A A A A A    
501	. G G G G G    
502	. G G G G G    
503	. A A A A A    
504	. G G G G G    
505	. A A A A A    
506	. C C C C C    
507	. G G G G G    
508	. A A A A A    
509	. G G G G G    
510	. A A A A A    
511	. T T T T T    
512	. G G G G G    
513	. G G G G G    
514	. A A A A A    
515	. G G G G G    
516	. A A A A A    
517	. A A A A A    
518	. A A A A A    
519	. A A A A A    
520	. A A A A A    
521	. G G G G G    
522	. A A A A A    
523	. A A A A A    
524	. G G G G G    
525	. C C C C C    
526	. C C C C C    
527	. G G G G G    
528	. A A A A A    
529	. A A A A A    
530	. T T T T T    
531	. G G G G G    
532	. C C C C C    
533	. C C C C C    
534	. A A A A A    
535	. G G G G G    
536	. T T T T T    
537	. A A A A A    
538	. T T T T T    
539	. T T T T T    
540	. G G G G G    
541	. T T T T T    
542	. T T T T T    
543	. T T T T T    
544	. A A A A A    
545	. A A A A A    
546	. G G G G G    
547	. A A A A A    
548	. T T T T T    
549	. A A A A A    
550	. A A A A A    
551	. A A A A A    
552	. G G G G G    
553	. C C C C C    
554	. A A A A A    
555	. C C C C C    
556	. T T T T T    
557	. G G G G G    
558	. T T T T T    
559	. G G G G G    
560	. A A A A A    
561	. T T T T T    
562	. A A A A A    
563	. T T T T T    
564	. G G G G G    
565	. A A A A A    
566	. G G G G G    
567	. C C C C C    
568	. C C C C C    
569	. A A A A A    
570	. T T T T T    
571	. T T T T T    
572	. C C C C C    
573	. G G G G G    
574	. A A A A A    
575	. G G G G G    
576	. C C C C C    
577	. C C C C C    
578	. C C C C C    
579	. T T T T T    
580	. A A A A A    
581	. T T T T T    
582	. A A A A A    
583	. T T T T T    
584	. T T T T T    
585	. A A A A A    
586	. G G G G G    
587	. G G G G G    
588	. T T T T T    
589	. G G G G G    
590	. A A A A A    
591	. T T T T T    
592	. T T T T T    
593	. G G G G G    
594	. T T T T T    
595	. G G G G G    
596	. G G G G G    
597	. C C C C C    
598	. T T T T T    
599	. G G G G G    
600	. T T T T T    
601	. T T T T T    
602	. A A A A A    
603	. G G G G G    
604	. A A A A A    
605	. A A A A A    
606	. C C C C C    
607	. A A A A A    
608	. A A A A A    
609	. A A A A A    
610	. A A A A A    
611	. T T T T T    
612	. C C C C C    
613	. A A A A A    
614	. A A A A A    
615	. T T T T T    
616	. C C C C C    
617	. T T T T T    
618	. G G G G G    
619	. T T T T T    
620	. A A A A A    
621	. T T A T T    
622	. A A A A A    
623	. T T T T T    
624	. T T T T T    
625	. C C C C C    
626	. A A A A A    
627	. A A A A A    
628	. A A A A A    
629	. T T T T T    
630	. G G G G G    
631	. T T T T T    
632	. A A A A A    
633	. A A A A A    
634	. G G G G G    
635	. C C C C C    
636	. C C C C C    
637	. T T T T T    
638	. C C C C C    
639	. T T T T T    
640	. G G G G G    
641	. T T T T T    
642	. A A A A A    
643	. A A A A A    
644	. T T T T T    
645	. A A A A A    
646	. T T T T T    
647	. T T T T T    
648	. G G G G G    
649	. T T T T T    
650	. A A A A A    
651	. C C C C C    
652	. T T T T T    
653	. T T T T T    
654	. C C C C C    
655	. A A A A A    
656	. G G G G G    
657	. T T T T T    
658	. T T T T T    
659	. T T T T T    
660	. T T T T T    
661	. A A A A A    
662	. T T T T T    
663	. G G G G G    
664	. T T T T T    
665	. T T T T T    
666	. . G G G G    
667	. . T T T T    
668	. . G G G G    
669	. . A A A A    
670	. . G G G G    
671	. . A A A A    
672	. . A A A A    
673	. . T T T T    
674	. . A A A A    
675	. . A A A A    
676	. . A A A A    
677	. . G G G G    
678	. . A A A A    
679	. . T T T T    
680	. . A A A A    
681	. . T T T T    
682	. . A A A A    
683	. . T T T T    
684	. . T T T T    
685	. . A A A A    
686	. . C C C C    
687	. . A A A A    
688	. . C C C C    
689	. . T T T T    
690	. . T T T T
```
